# Micro-/Nano-Structured Ceramic Scaffolds That Mimic Natural Cancellous Bone

**DOI:** 10.3390/ma14061439

**Published:** 2021-03-16

**Authors:** Anabel Díaz-Arca, Patricia Ros-Tárraga, María J. Martínez Tomé, Antonio H. De Aza, Luis Meseguer-Olmo, Patricia Mazón, Piedad N. De Aza

**Affiliations:** 1 Instituto de Bioingeniería, Universidad Miguel Hernández, 03202 Elche, Spain; a.diaza@umh.es (A.D.-A.); patricia.ros01@goumh.umh.es (P.R.-T.); pmazon@umh.es (P.M.); 2 Instituto de Investigación, Desarrollo e Innovación en Biotecnología Sanitaria de Elche, 03202 Elche, Spain; mj.martinez@umh.es; 3 Instituto de Cerámica y Vidrio, ICV-CSIC, 28049 Madrid, Spain; aaza@icv.csic.es; 4 Grupo de Investigación en Regeneración y Reparación de Tejidos, Universidad Católica San Antonio de Murcia, Guadalupe, 30107 Murcia, Spain; lmeseguer@ucam.edu

**Keywords:** biomimetic, cancellous bone, ceramic scaffold, micro-/nano-structure, tissue engineering

## Abstract

Micro-/nano-structured scaffolds with a weight composition of 46.6% α-tricalcium phosphate (α-TCP)—53.4% silicocarnotite (SC) were synthesized by the polymer replica method. The scanning electron microscopy (SEM) analysis of the scaffolds and natural cancellous bone was performed for comparison purposes. Scaffolds were obtained at three cooling rates via the eutectoid temperature (50 °C/h, 16.5 °C/h, 5.5 °C/h), which allowed the surface nanostructure and mechanical strength to be controlled. Surface nanostructures were characterized by transmission electron microscopy (TEM) and Raman analysis. Both phases α-TCP and SC present in the scaffolds were well-identified, looked compact and dense, and had neither porosities nor cracks. The non-cytotoxic effect was evaluated in vitro by the proliferation ability of adult human mesenchymal stem cells (ah-MSCs) seeded on scaffold surfaces. There was no evidence for cytotoxicity and the number of cells increased with culture time. A dense cell-hydroxyapatite layer formed until 28 days. The SEM analysis suggested cell-mediated extracellular matrix formation. Finally, scaffolds were functionalized with the alkaline phosphatase enzyme (ALP) to achieve biological functionalization. The ALP was successfully grafted onto scaffolds, whose enzymatic activity was maintained. Scaffolds mimicked the micro-/nano-structure and chemical composition of natural cancellous bone by considering cell biology and biomolecule functionalization.

## 1. Introduction

Scaffold-based tissue engineering is a discipline that employs templates to stimulate and support the bone regeneration process. An ideal scaffold should have a similar micro-/nano-structure and chemical composition to that in the bone mineral phase to provide a native environment. To improve scaffold-host tissue interactions, templates can be functionalized with several cells, proteins and growth factors [1,2]. Scaffolds should also promote osseointegration by means of bioactive, osteoinductive, and osteoconductive properties. Finally, they must have surface properties to improve vascular ingrowth and to allow cell fixation and proliferation [3,4].

Porosity is one of the most important factors to control when designing scaffolds because both microporosity (<10 µm pore size) and macroporosity (>100 µm pore size) play a key role in the cellular processes that promote bone growth [5]. Other involved factors are composition and the phases present in the material constituting scaffolds. Hence ceramic materials in the Si-Ca-P family, such as Si-hydroxyapatite (Si-HA), Si-tricalcium phosphate (Si-TCP), Nurse’s A phase (7CaOP_2_O_5_2SiO_2_) and silicocarnotite (SC-5CaOP_2_O_5_SiO_2_), mimic the behavior of natural apatites by forming an active chemical bond on its surfaces that leads to bone tissue neoformation [6,7]. The outstanding biocompatibility of such a ceramic is thanks to the chemical similarity to the bone mineral phase, which is constituted by about 70% calcium phosphate known as hydroxyapatite [8].

A range of methods and materials has been indicated to synthesize and process these ceramics, including biological and synthetic sources and adding ions to improve their biological responses and other properties [9,10,11,12]. Here bioinspired micro-/nano-structured ceramic scaffolds sintered by a polyurethane replica technique based on calcium, phosphorus and silica are presented that can act as scaffolds for bone tissue engineering. Given the different behaviors of its constituent phases, this material develops lamellar micro-/nano-porosity when exposed to a physiological medium. To obtain micro-/nano-porosity, a distinctive composition in the TCP-SC subsystem was selected [13]. This composition was that which corresponded to the invariant eutectoid point because a lamellar structure developed during cooling [14]. In order to control the surface nanostructures and mechanical strength, the sintering process involved a new heat treatment variant, including radial heat extraction, cooling rate control via the eutectoid temperature and low cooling until room temperature.

A comparative SEM study between the bioinspired new materials and natural cancellous bone was performed to evaluate ultrastructural similarity. To estimate the scaffolds’ non-cytotoxic effect, cell attachment and proliferation ability were studied in vitro. An enzyme involved in the bone formation and mineralization process (alkaline phosphatase, ALP) was grafted to scaffolds to achieve biological functionalization.

## 2. Materials and Methods

### 2.1. Ceramic Scaffolds Synthesis

SC and α-TCP powders were used as raw ceramic materials to synthesize eutectoid lamellar scaffolds following the previously reported sintering and characterization process [15]. Briefly, 53.4 wt% SC and 46.6 wt% α-TCP powder was ground to an average particle size of 12 µm (Mastersizer 2000, Malvern Instruments Ltd., Malvern, PA, USA). The barbotine suspension of grinding powder was prepared to coat the polyurethane templates (ppi 30) in the macroporous green samples prior to a sintering process run in a 50 mL platinum foil crucible at a heating rate of 85 °C/h in an Entech electric furnace, which was raised to 1550 °C/3 h. In order to control the surface nanostructure and mechanical strength, from this temperature three cooling rates were studied through the eutectoid temperature (50 °C/h, 16.5 °C/h and 5.5 °C/h). At 1100 °C, the cooling rate was changed back to 85 °C/h and cooling continued until room temperature. The manufactured scaffolds were cylinders (8 mm diameter × 4 mm high).

### 2.2. Scaffolds Characterization

Scaffolds’ volumetric shrinkage S_v_ (%) was assessed in 10 specimens for each cooling rate as:

S_v_ = (1 − V_s_/V_0_) × 100% (1)

where V_s_ is the scaffold final volume and V_0_ is the volume of the green body before heat treatment. Open porosity (%) (pores larger than 100 μm) was evaluated by Archimedes’ method. Scaffolds’ strength, in terms of maximum compressive stress before fracture, was tested by the crushing test in a Simple Manual Test Stand (NEURTEK instruments SVL-1000N). Axial force was constantly applied to scaffolds until fracture, and was recorded in a digital force gauge dst/dsv SERIES. Maximum compressive stress σ_c_ (MPa) was calculated as:

σ_c_ = F_c_/A (2)

where F_c_ (N) is the maximum force recorded during the test and A (mm^2^) is the scaffolds’ cross-sectional area perpendicular to axial force. Ten specimens for each cooling rate were tested. Results were represented by mean values ± standard deviation.

The scaffolds’ micro-/nano-structure was characterized by scanning electron microscopy in a SEM-Hitachi S-3500N device with an Energy-Dispersive X-Ray Spectroscopy (EDS-INCA, Oxford Instruments Analytical, Oxford, UK) using a JEOL JEE-400 vacuum evaporator to coat specimens with palladium. The SC-TCP lamellae interphase on scaffold surfaces was studied in more detail by a transmission electron microscopy (TEM-HRTEM-JEM-2010 Jeol Ltd., Tokyo, Japan) analysis. To determine the Raman active vibration modes of phosphate-silicate tetrahedra stretching and bending, Raman spectra were recorded at room temperature using a Witec ALPHA 300RA Confocal Raman device within the 200–1200 cm^−1^ frequency ranges, and with 532 nm Nd: YAG laser light source in the p-polarization mode and a 20× objective lens.

### 2.3. SEM Examination of Natural Cancellous Bone

The cancellous bone ultrastructure analysis was performed by the SEM examination of adult pig distal femoral epiphysis in a JEOL JSM 6100 that operated at 15–20 kV. Specimens were obtained in a sagittal section from the epiphyseal plate using a 10 mm trephine (Shanghai LZQ-Technology, Shanghai, China). Bone samples, initially cut with a cylindrical morphology of 10 mm in diameter and 10.5 mm thickness, were sectioned perpendicularly to the longitudinal axis in discs that were 1.5 ± 0.3 mm thick. Specimens were cleaned to remove all types of organic matter by immersing them in H_2_O_2_ with continuous magnetic stirring for 12 h, and then in 10% Triton X-100 detergent solution (Merck, Darmstadt, Germany) for 24 h. Finally, they were washed with deionized water for 24 h and dried at 37 °C. For the SEM examination, the extracellular matrix structures were processed as indicated below:

- Fixation for 2.5 h in a McDowell’s and Trump’s 4F:1G fixative solution [16] that consists in a combination of 4% commercial formaldehyde and 1% glutaraldehyde in a buffer of 176 mOsm/liter. It is recommended as a primary fixative for the SEM analysis of samples.
- Washed in cacodylate buffer at 0.1 Μ and saccharose at 8% overnight at 4 °C.
- Fixation was completed with 1% osmium tetroxide and 0.1 Μ cacodylate for 1.5 h at 4 °C.
- Dehydration by gradient acetone series 30%, 50%, 70%, 90%, and 100% for 10 min each at room temperature
- Critical point drying in CO_2_.
- Finally, samples were gold-coated by the Bio-Rad Polaron coater (Bio-Rad Laboratories, Hercules, CA, USA).

After removing organic bone elements, many evident images of the mineralized matrix were obtained.

### 2.4. Ah-MSCs: Isolation, Expansion and Characterization

Undifferentiated multipotent adult human mesenchymal stem cells (ah-MSCs) were isolated from the human bone marrow according to a previously reported protocol [17]. The previously signed informed consent of volunteers was needed for the intervention. The assay protocol was approved for the Institutional Ethics Committee of the Universidad Católica San Antonio (authorized nºCE051904). Ah-MSCs were characterized according to the minimal standard criteria established by Dominici et al. [18]. The methods followed for the culture and expansion of ah-MSCs are found in a previous work [19]. Expanded cells, passage 3 (P3), were collected for the in vitro tests.

### 2.5. Ah-MSCs Adhesion and Proliferation In Vitro Test

In adhesion and proliferation terms, the non-cytotoxic effect of scaffolds on ah-MSCs was studied in vitro on scaffold surfaces. The scaffold cooled at 5.5 °C/h was selected for the assay as it exhibited improved mechanical strength. Two kinds of cell culture media were used:

(a) Basal growth medium (GM). It consists in 10% inactivated fetal bovine serum (FBS) and 1% antibiotics (penicillin (100 U/mL)/streptomycin (100 µg/mL)) supplemented in Dulbecco’s Modified Eagle Medium (DMEM) (Sigma-Aldrich, St. Louis, MO, USA).
(b) Osteogenic-inducing medium (OM). It consists in GM with an osteogenic supplement composed of L-ascorbic acid 2-phosphate (0.2 mM; mSigma, St. Louis, MO, USA), dexamethasone (10 nM; Sigma) and β-glycerolphosphate (10 mM; Merck, Darmstadt, Germany).

Sterilized scaffolds were incubated in GM for 1 h before seeding. Cells were seeded at a density of 5 × 10^3^ cells/cm^2^ on scaffold surfaces in 48-well plates. Incubation was performed at 37 °C, 5% CO_2_, and 95% relative humidity with 800 μL of GM for 7, 14, 21, and 28 days according to previous works [17,19]. The cells grown in plastic, in the absence of scaffolds, were used as a positive control. At 21 days, half the samples were changed from GM to OM until 28 days. After each incubation time, cell metabolic functions were assessed by the Alamar Blue assay. Samples were washed twice with phosphate buffer saline 1× (PBS 1×) and supplied with 800 μL of GM with 10% Alamar Blue (Invitrogen, Carlsbad, CA, USA). Then, samples were incubated for 4 h in the dark at 37 °C. After 4 h, 200 μL of reactant were placed in a new 96-well plates for the fluorescence analysis in a Synergy MX ultraviolet visible (UV–Vis) reader (BioTeK Instruments Inc., Winooski, VT, USA). The excitation-emission wavelengths were 560–590 nm, respectively. For the SEM examination, the cell-cultured scaffolds were rinsed for 10 min in PBS 1x and fixed with 3% glutaraldehyde for 1 h. Samples were preserved in cacodylate buffer (0.1 Μ) until fixation with 1% osmium tetroxide. They were dehydrated in a gradient series of ethanol solutions (30%, 50%, 70%, 90%, and 100% *v/v*) and critical point drying with liquid CO_2_. Finally, samples were palladium-coated for the SEM examination.

### 2.6. Testing Alkaline Phosphatase Specific Activity in Cells

ALP activity is an early marker of osteogenic differentiation. The influence of scaffolds’ material on the initial differentiation of ah-MSCs was estimated by ALP specific activity. The cells seeded at a density of 5 × 10^3^ cells/cm^2^ in 48-well plates were cultured in indirect contact with scaffolds using 800 μL of GM for 14 and 28 days, and OM for 28 days (37 °C, 5% CO_2_, and 95% relative humidity). ALP activity was assessed by the reaction whit the substrate p-nitrophenyl phosphate (PNPP) (Sigma). Under optimal conditions, PNPP is hydrolyzed by the ALP to result in p-nitrophenol (PNP), characterized by a yellow color. The amount of resulting PNP is proportional to ALP activity and can be quantified by Vis-spectroscopy. Measures were taken at 405 nm by a Synergy MX ultraviolet visible (UV–Vis) reader (BioTeK Instruments Inc., Winooski, VT, USA).

### 2.7. Alkaline Phosphatase Grafting to Scaffolds

In order to biofunctionalize scaffolds, the ALP was grafted onto their structures. The scaffold cooled at 5.5 °C/h was selected for the assay as it exhibited improved mechanical strength. The ALP was chosen as the enzyme model because its activity plays an active role in bone formation and mineralization processes [20,21]. To prepare ALP solution (250 µΜ), bovine ALP (from bovine intestinal mucosa, lyophilized powder, ≥10 DEA units/mg solid, Sigma–Aldrich) was dissolved in Tris (hydroxymethyl) aminomethane ((HOCH_2_)_3_CNH_2_, 99.9+% ultrapure grade, Sigma–Aldrich) buffer (55 mΜ, pH 9) with ultrasonic stirring.

Three scaffolds were immersed in 1 mL of ALP solution for 24 h at 4 °C. When incubation ended, samples were rinsed in 5 mL of Tris buffer with magnetic stirring for 10 min and then dried at room temperature. Finally, they were stored hydrated in Tris buffer in a Petri dish at 4 °C. The functionalized samples’ effectiveness was investigated by running an enzymatic activity test as described below.

### 2.8. Enzymatic Activity Test

The ALP grafted onto scaffolds should be biologically active to achieve suitable enzymatic functionalization. Enzyme activity was studied by the reaction between the grafted ALP and substrate PNPP. In order to determine grafted ALP activity after 7, 14, 21 days of functionalization, samples were placed in 5 mL of the 50 μΜ PNPP (Fisher Scientific, Fair Lawn, NJ, USA) solution in Tris buffer. The reaction between the ALP grafted onto scaffolds and PNPP was quantified by measuring absorbance at 405 nm in a UV-2700 Spectrophotometer (Shimadzu, Tokyo, Japan) every 40 min for 2 h. At the end of each test, samples were rinsed 3 times in 5 mL of Tris buffer with magnetic stirring for 10 min. Then they were stored hydrated in Tris buffer in a Petri dish at 4 °C until the next test.

### 2.9. Statistical Analysis

Data are represented by mean ± standard deviation. Anova was used to analyze the variance between different groups. The *p*-value denotes statistically significant differences when *p* < 0.05.

## 3. Results

### 3.1. Scaffolds’ Characterization and Cancellous Bone Examination

**Figure 1:**
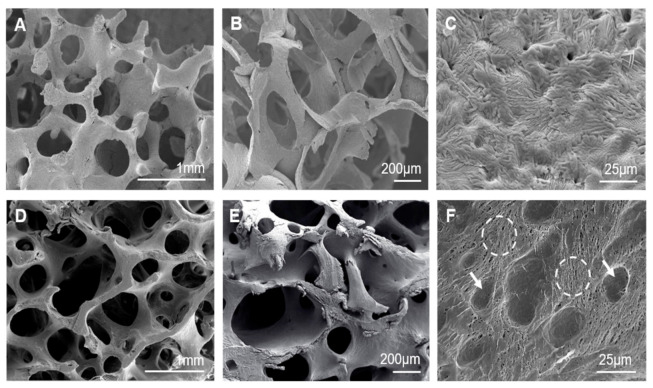
SEM micrographs at different magnifications showing the micro-/nano-structure of: (**A**–**C**) the eutectoid lamellar scaffold cooled at 50 °C/h as being a representative sample of them all and (**D**–**F**) natural cancellous bone.

After each heat treatment, all the scaffolds showed a 30% volumetric shrinkage average (Table 1). The SEM examination proved that, despite shrinkage, all scaffolds continued to maintain a highly interconnected 3D microstructure with a pore size range of 150–600 µm and an open porosity of 85 ± 2% (Figure 1A,B).

The nanostructure of scaffold surfaces was seen in detail after being etched for 4s with 1% dilute acetic acid (Figure 1C). Scaffold nanostructures came in the form of alternating lamellae, which suggests an irregular eutectoid structure that is homogeneously distributed over the entire surface of pore walls and struts. After chemical etching, EDS indicated the presence of calcium, phosphorous and silicon at a ratio 1.34 < Ca/(P+Si) < 1.88. On average, this value came close to that of stoichiometric silicocarnotite (Ca/(P+Si) = 1.66) which suggests that α-TCP could be the phase eliminated by chemical etching. The results from mechanically testing scaffolds are displayed at Table 1. Scaffold strength, in terms of maximum compressive stress before fracture, increased as the cooling rate lowered. The scaffold cooled at 5.5 °C/h reached a mechanical strength within the range for cancellous bone (2–12 MPa) [22].

The SEM examination of the natural cancellous bone microstructure showed a highly interconnected trabeculae network with heterogeneous distributions of macropores, whose pore size range was 100–700 µm and an open porosity of 87 ± 1% (Figure 1D,E). EDS confirmed the presence of calcium and phosphorous at a ratio of 1.50 < Ca/P < 1.60.

The nanoscale surface topography of natural cancellous bone (Figure 1F), which resulted from removing the bone organic component, showed two well-defined areas: resorbing and resting surfaces. Resorbing surfaces (the arrows in Figure 1F) were identified as resorption bays, known as Howship’s lacunae, produced by osteoclast erosion. Resting surfaces (the circles in Figure 1F) were identified as lamellae that alternated in a random orientation. This morphology corresponds to the pattern of the collagen fiber bundles previously removed by sample cleaning processes. This observation agrees with the SEM data for cancellous bone reported by other authors [23,24,25]. Both the micro-/nano-structure of the sintered scaffolds came close to that of examined natural cancellous bone.

### 3.2. Characterization of the Nanostructure of Scaffold Surfaces

**Figure 2:**
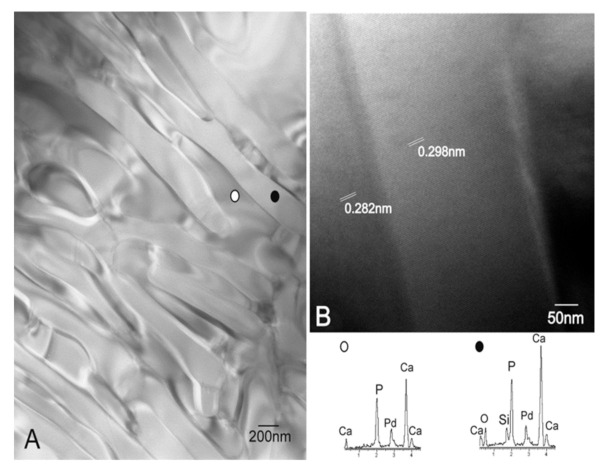
(**A**) Representative TEM image of the eutectoid scaffold surfaces and (**B**) the HRTEM image of the α-TCP-SC lamellae interphase and the EDS analysis. The scaffold cooled at 50 °C/h was representative of all the scaffolds.

Figure 2 shows representative TEM images of scaffold surfaces (scaffold cooled at 50 °C/h as being representative of all the scaffolds). Figure 2A illustrates an irregular alternating lamellae nanostructure, which confirmed the presence of two phases in scaffolds. The EDS analysis displayed a silicon-rich phase (● Figure 2) that corresponded to the SC phase and a calcium-phosphate phase (○ Figure 2) that corresponded to the α-TCP phase. Both phases look compact and dense with neither porosities nor cracks.

The HRTEM examination of the interfaces between SC and α-TCP (Figure 2B) indicated a smooth continuous morphology between both phases. There was no intermediate region among the lamellae up to the lattice plane resolution level. No resolved lattice plane was interrupted up to the interface, and they were all free of defects. The lattice fringes associated with d = 0.298 nm corresponded to a preferred (034) orientation, as determined in Figure 2B, which confirmed that the lighter phase was α-TCP and the darker phase was SC with d = 0.282 nm corresponding to a preferred (230) orientation.

**Figure 3:**
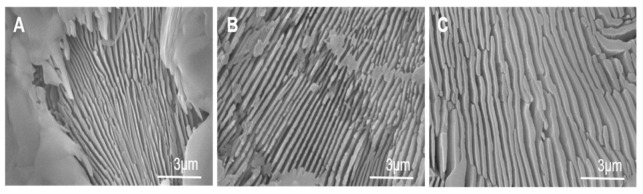
SEM micrographs of scaffold surfaces after chemical etching with acetic acid for a cooling rate of: (**A**) 50 °C/h (**B**) 16.5 °C/h and (**C**) 5.5 °C/h.

A more detailed SEM analysis of scaffold surfaces, after being etched for 4s with 1% dilute acetic acid (Figure 3), showed that lamellae thickened as the cooling rate lowered to give a lamellar width range of 100–250 nm, 300–560 nm and 600–940 nm for the cooling rate of 50 °C/h, 16.5 °C/h, and 5.5 °C/h, respectively (Table 1). Therefore, it is possible to control the eutectoid nanostructure on scaffold surfaces with the cooling rate of the sintering process.

**Figure 4:**
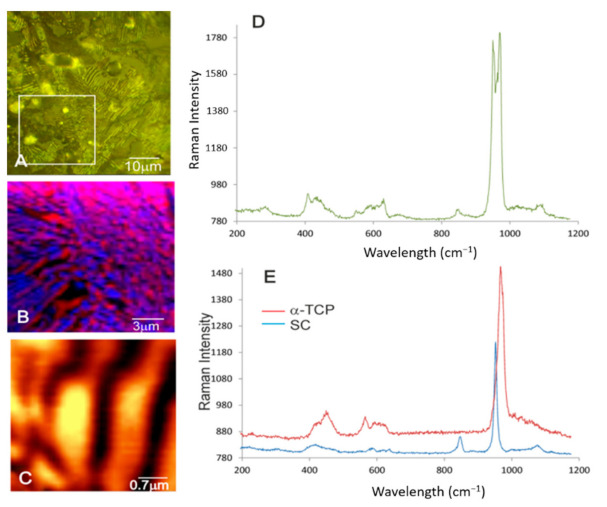
(**A**) Confocal optical micrograph of the scaffold cooled at 50 °C/h as being a representative sample. (**B**) Raman image of the area selected in (**A**) in which phases SC and α-TCP are represented in colors and display an irregular eutectoid pattern. (**C**) A Rayleigh light-scattering microscopy image showing a nanostructured sinusoidal disordered pattern. (**D**) The average Raman spectra from the scaffold. (**E**) Single-phase dense ceramics of SC and α-TCP for comparison purposes.

Figure 4 displays the confocal optical micrographs of the scaffold cooled at 50 °C/h as being a representative sample of all the scaffolds. A similar lamellar nanostructure to that observed in the SEM images in Figure 1C and Figure 3 is seen. Raman scanning was performed in the marked selected square in Figure 4A, which allowed a colored Raman image to be obtained with phase distribution (Figure 4B). Two eutectoid composition phases can be discriminated: SC and α-TCP. The average Raman spectrum of phases SC and α-TCP are shown in Figure 4D. For comparison purposes, Raman spectra were also obtained for two single phase SC and α-TCP dense ceramics manufactured in our laboratory (Figure 4E) [7,26].

The Raman spectrum of SC displayed the following bands (cm^−1^): 302 and 234 ν(Ca-O); 420 ν_2_(SiO_4_); 470 ν_2_(PO_4_); 580 [ν_2_(PO_4_) + (SiO_4_)]; 640 ν_4_(PO_4_); 850 ν_1_(SiO_4_); 956 ν_1_(PO_4_); 1000 ν_1_(SiO_4_); 1080 [ν_3_(PO_4_) + ν_3_(SiO_4_)] [7,27]. The strongest bands were assigned to the vibrations of (SiO_4_) and (PO_4_) tetrahedra.

The Raman spectrum of α-TCP displayed the following bands (cm^−1^): 439, 460, 475, 483 ν_2_(PO_4_); 565, 578, 588, 599, 611, 624 ν_4_(PO_4_); 960, 970 ν_1_(PO_4_); 1005, 1016, 1031, 1046, 1060, 1075, 1085, 1090 ν_3_(PO_4_) [28,29]. The Raman spectrum of the scaffold was separated into six groups of bands as follows (cm^−1^): 256–300; 425–467; 551–640; ~844; 950–970; 1000–1096.

Overlapping took place with the (PO_4_) and (SiO_4_) groups from SC and α-TCP. Isolated peak was distinguished at 845 that belonged to ν_1_(SiO_4_) from SC and a triple peak at 956-976-970, which allowed to distinguish ν_1_(PO_4_) from SC, and ν_1_(PO_4_) from α-TCP.

A mixture of SC and α-TCP phases was identified in the average Raman spectrum of the analyzed scaffold regions. In an attempt to distinguish both phases, Rayleigh light-scattering was carried out to verify the different phase compositions of the lamellae in the scaffold. Figure 4C depicts a Rayleigh image that was taken from a lamellar micrograin. It reveals a striped pattern with bright and dark areas, where yellow denotes the SC phase and dark areas depict the α-TCP phase.

### 3.3. Cell adhesion and Proliferation In Vitro Test

**Figure 5:**
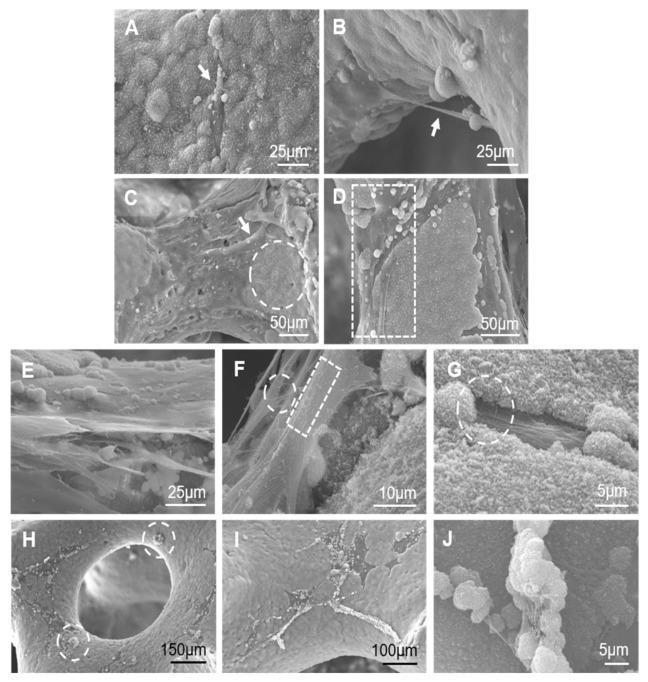
SEM micrographs at different magnifications of scaffold surfaces seeded with ah-MSCs in GM for: (**A**,**B**) 7, (**C**–**G**) 14 and (**H**–**J**) 28 days.

The scaffold cooled at 5.5 °C/h was evaluated in terms of the adhesion and proliferation ability of the ah-MSCs seeded on their surfaces. This scaffold was selected for the assay as it exhibited improved mechanical strength. The SEM micrographs of ah-MSCs’ evolution on scaffold surfaces are shown in Figure 5 for different incubation times in GM. After seven days (Figure 5A,B), the agglomeration of a spherical Ca-P precipitate formed a dune-like layer that covered scaffold surfaces. Individual cells were observed to adhere to scaffold surfaces by means of extensive cytoplasmic projections (the arrows in Figure 5A,B). For 14 days after seeding (Figure 5C–G), flattened and completely adhered cells (the arrow in Figure 5C) formed isolated groups and came into close physical contact with one another and the scaffold surfaces through numerous cytoplasmic projections. At this time, some cell-free areas were still visible on scaffold surfaces where the typical lamellar nanostructure was distinguished (the circle in Figure 5C).

Moreover, some cells formed a monolayer adhered on the dune-like precipitate and, at the same time, a new dune-like precipitate was formed above the cell monolayer in close contact with their membranes (Figure 5D). The rectangle in Figure 5D shows some cells spreading onto the new precipitate, which suggests the formation of a layer-by-layer structure. Figure 5E shows a cross-sectional view where the dune-like precipitates were observed in the background and at the top of the cell monolayer.

Figure 5F shows some processes in detail that took place on the periphery of cell membranes. A basal process, with many exocytosis vesicles (the rectangle in Figure 5F), was observed and their content was assembled to form the collagen-like fibrils observed around cell membranes (the circle in Figure 5F). This collagen-like meshwork initially took a random spatial orientation and was integrated with the dune-like precipitate (the circle in Figure 5G). For 21 days, the SEM observation showed a similar behavior on scaffold surfaces (data not shown). By prolonging the incubation time to 28 days, the layer-by- layer cell and dune-like deposition precipitate formed a dense and almost complete layer over the entire scaffold surfaces (Figure 5H,I). SEM revealed that this monolayer looked granular with some superficial protrusions (the circles in Figure 5H).

The presence of a fibrillary meshwork together with Ca-P mineral deposits suggests the initial formation of mineralization nodules at these superficial protrusions (see the detail in Figure 5J).

**Figure 6:**
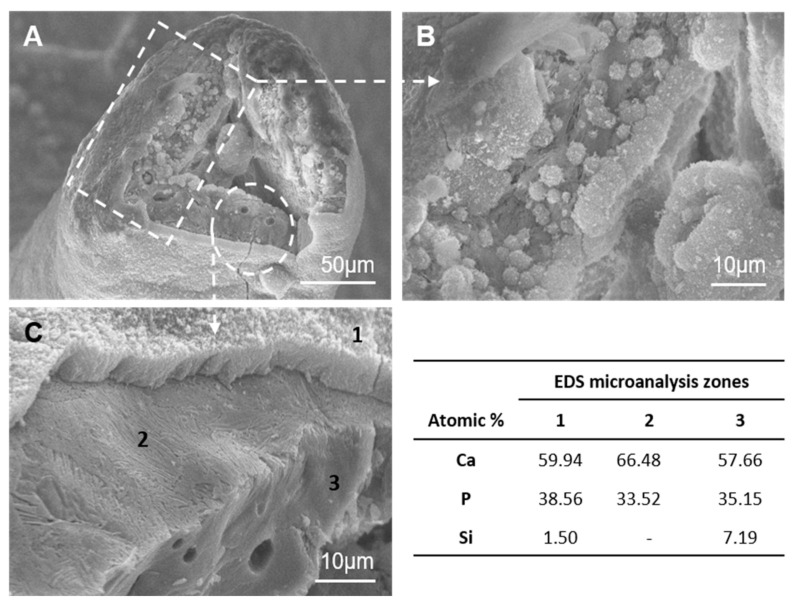
SEM micrographs at different magnifications of a broken scaffold strut after 28 days in GM with ah-MSCs: (**A**) the broken strut, (**B**) detail of a globular niche produced after collagen-like fibril formation and (**C**) detail of three well-defined consecutive structures.

Figure 6 provides details of a broken scaffold strut, after 28 days in GM with ah-MSCs. The ultrastructural analysis revealed two different zones on the strut boundary. The layer- by- layer cell and dune-like precipitate deposition was identified (the rectangle in Figure 6A). The detail in Figure 6B shows a globular niche produced after collagen-like fibril formation that corresponds to the previously reported SEM observations for the cell-mediated extracellular matrix formation during the biomineralization process [30,31]. Moreover, a cell-free zone was also detected (the circle in Figure 6A). The magnification in Figure 6C showed three well-defined consecutive structures:

(1) A dense dune-like layer on the GM-scaffold boundary with an average Ca/P ratio of 1.55. EDS indicated less silicon than the original sample (3). This layer was about 9 ± 0.05 µm thick. The crystalline nature of this dune-like layer and the Ca/P ratio corresponded to the precipitated calcium-deficient nonstoichiometric hydroxyapatite.
(2) A lamellar microstructure lay beneath the precipitated hydroxyapatite layer with an average Ca/P ratio of 1.98. EDS confirmed that the silicon was not present in the lamellar microstructure.
(3) The original scaffold material remained inside the structure with an average Ca/P ratio of 1.64. EDS indicated higher silicon content than the precipitated hydroxyapatite layer.

**Figure 7:**
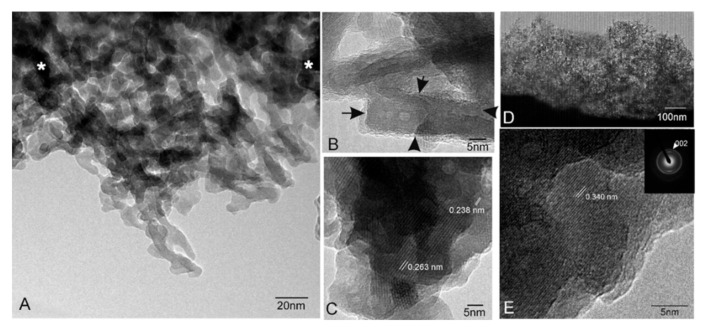
TEM images of the scaffold surfaces’ product: (**A**) after 7 days in culture (* dark regions from where the crystals grew), (**B**) zoom into the rectangular crystals, (**C**) the HRTEM image of the region, (**D**) after 28 days in culture showing the morphology of the new precipitate Ca-P phase and (**E**) the HRTEM of the region with a SAD pattern.

The TEM-HRTEM analysis was performed to study the changes in the hydroxyapatite layer in more detail after incubation times. After seven days in culture, elongated crystals were observed whose lengths ranged between 15 and 150 nm (Figure 7A). These growing crystals were seen to grow from dark regions (* in Figure 7A). The formation of amorphous calcium phosphate clusters, associated with dark areas, has been reported by Liao et al. [32] and Dey et al. [33], who described the geometry of crystals as a “parallel array of needles” and their size with CO_3_^2−^ content. Figure 7B shows several overlapped crystals in which a single one was approximately 12 nm wide and 20 nm long. Finally, an HRTEM image (Figure 7C) shows a higher density of crystals joined with lattice fringes between them (0.238 < d < 0.263 nm).

After 28 days, a significant density of needle arrays was seen (Figure 7D), where the Ca/P ratio of ~1.55 evidenced the formation of calcium-deficient apatite, which is usually found in bioactive materials [6,10,34]. In this phase, these needle–like crystals had completely merged to form a homogeneous layer with practically no space left between nanocrystals. It is noteworthy that the morphology and composition of crystals observed by TEM coincided with the growth of the needle-like crystals that formed the globular precipitated observed by SEM after seven days in culture (Figure 5A). The compositional gradient found in the EDX analysis of the cross-sectional view confirmed the TEM results. It revealed calcium-deficient apatite with Si contents. The HRTEM performed of the mature crystallized apatite layer is found in Figure 7E, where a higher density of joined crystals was observed with lattice fringes of d = 0.340 nm.

There was no evidence for either cytotoxicity or cell morphological alterations throughout the observational SEM analysis. The number of cells on scaffold surfaces increased with culture time. The hydroxyapatite layer did not obstruct cell adhesion and proliferation.

**Figure 8:**
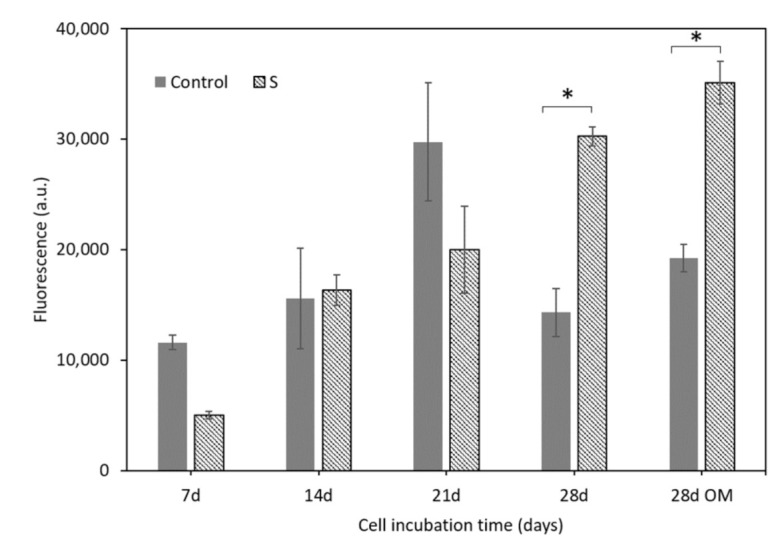
Metabolic activity of the ah-MSCs seeded on scaffold surfaces (S) and the positive control (Control) after different culture times in GM and OM. * denotes significant differences (*p* < 0.05) between them at the same culture time.

Cell proliferation was quantified by the Alamar Blue assay whose results confirmed the SEM observation. Figure 8 shows metabolic activity of the ah-MSCs seeded on scaffold surfaces (S) and on the 2D tissue culture polystyrene plates as the positive control (Control) after different culture times in GM and OM.

Cell metabolic activity on scaffold surfaces progressed linearly with time until the end of the assay and its value overtook that of the control after 28 days. At this time, the low absorbance value of the control was associated with cell confluence. On polystyrene plates, cells grew in a 2D environment and proliferation was restricted by the available surface area, cell-cell interactions and nutrient availability on the cell-GM/OM boundary. Scaffolds’ 3D structure and porosity provided a bigger surface area for cell proliferation and a more efficient oxygen and nutrients exchange. The cells seeded on scaffold surfaces showed significant differences in metabolic activity at 28 days compared to the positive control (*p* < 0.05).

### 3.4. Specific Alkaline Phosphatase Activity in Cells

**Figure 9:**
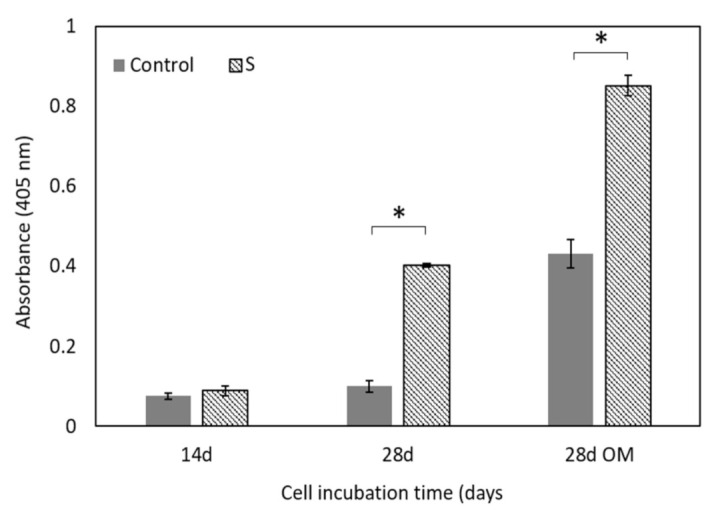
The quantification of ALP activity of the ah-MSCs seeded on scaffold surfaces (S) and the positive control (Control) after different culture times in GM and OM. * denotes significant differences (*p* < 0.05) between them at the same culture time.

Cell differentiation was estimated in terms of the ALP activity of the ah-MSCs cultured in scaffolds (S) and the positive control (Control), after 14 and 28 days in GM and after 28 days in OM (Figure 9).

After 14 days in GM, ALP activity was expressed at low levels and no significant differences were detected between scaffolds and the positive control. After 28 days in GM and OM, the ALP activity in the ah-MSCs incubated with the scaffold surfaces was significantly higher than in the control.

### 3.5. Alkaline Phosphatase Grafting to Scaffolds

**Figure 10:**
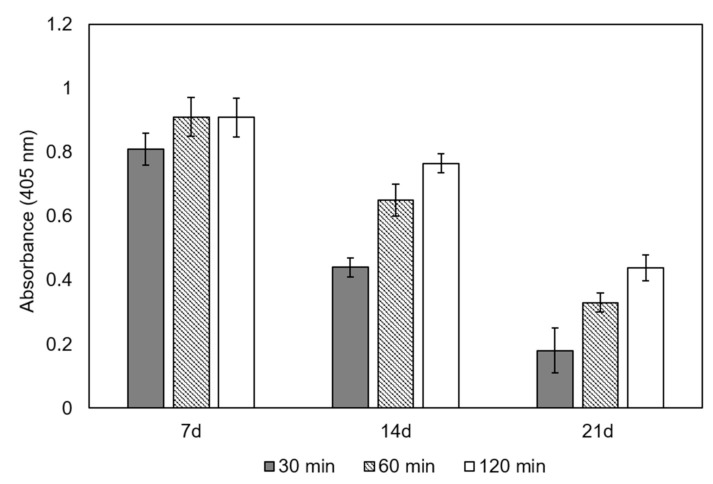
Specific ALP activity in scaffolds at 7, 14 and 21 days after functionalization. The enzyme activity kinetics was quantified after 40, 80 and 120 min of the reaction between the grafted ALP and PNPP solution.

The specific ALP activity on scaffolds at 7, 14, and 21 days after functionalization is shown in Figure 10. Enzyme activity was quantified by measuring absorbance at 405 nm after 40, 80 and 120 min of the reaction between the grafted ALP (250 µΜ) and PNPP (50 µΜ) solution. After 7 days of functionalization, the ALP grafted onto scaffolds transformed all the PNPP at 80 min. Therefore, no difference was detected between the 80 min and 120 min measurements. This indicated that ALP was successfully grafted onto scaffolds and its active state remained. Although enzymatic activity increased with the reaction time between ALP and PNPP for the same test, it should be noted that ALP activity decreased from one test to the next. As samples were washed 3 times in Tris buffer with magnetic stirring for 10 min after each test, the grafting stability between ALP and scaffolds could be involved in this result. In this case, a fraction of grafted ALP could be released to the washing solution.

## 4. Discussion

Nowadays, biomimetics is an essential issue for designing scaffolds whose aim is bone tissue regeneration. To provide a natural environment, an ideal scaffold must not only mimic the structure and chemical composition of natural bone, but should also consider cell biology and biomolecules as much as possible. In this context, eutectoid lamellar scaffolds were developed in the present work to mimic the micro-/nano-structure of natural cancellous bone, as well as the biomineralization process that would take place through the nucleation and growth of hydroxyapatite crystals on scaffold surfaces. Cells and protein functionalization were also supported in vitro.

Lamellar scaffolds were manufactured using a polyurethane template coated with a barbotine from the eutectoid ceramic composition corresponding to the TCP-SC subsystem, followed by solid-state reaction sintering [13]. Combining these methods is nothing new to other researchers, who have obtained porous scaffolds with future tissue engineering applications [2,5,8]. In the present work however, a new variant was included in the manufacturing process of scaffolds: using a unique singular composition of the system, such as a eutectoid composition, with slow cooling and radial heat extraction from the ceramic sample, to obtain a scaffold with a structure of alternating SC and α-TCP lamellae. The microstructural analysis of scaffolds showed a highly interconnected struts network enclosing large macropores similar to that observed for natural cancellous bone (Figure 1). Heterogeneous distributions of macropores were observed whose average size exceeded of 150 µm, which is reported as the minimum pore size needed to allow cell infiltration [35,36]. The larger pore size (>300 µm) improves vascularization, nutrient diffusion, and new tissue ingrowth [37]. Open porosity was about 85% within the range reported for cancellous bone (50–90%) [38] and came close to the value observed for an adult pig bone specimen (~87%).

The nanostructure of scaffold surfaces mimicked the rough surface of natural cancellous bone. The lamellar topography provided additional porosity to scaffolds and was able to improve cell adhesion and proteins adsorption. Furthermore, inter-lamellar gaps can act as a possible site for collagen fibers organization and subsequent bone matrix mineralization. Lamellae thickened as the cooling rate lowered, with a lamellar width range between 100–250 nm, 300–560 nm, and 600–940 nm for a cooling rate of 50 °C/h, 16.5 °C/h, and 5.5 °C/h respectively (Table 1). This was related to the way in which lamellae nucleated and grew according to the cooling rate [39]. Under our study conditions, at a slow cooling rate, the results of the nucleation of a few lamellae, led to lamellae becoming considerably coarser. Therefore, nanostructures can be controlled during the synthesis process by adjusting the cooling rate.

A detailed analysis of the eutectoid lamellar nanostructure by SEM, Raman, and TEM examinations showed a well-defined mixture of alternating SC and α-TCP lamellae over the entire scaffold surfaces. The interface among lamellae was free of defects, with no intermediate regions between them.

It was also possible to control the compressive strength by the cooling rate. The compressive strength increased as the cooling rate lowered (Table 1). It is well known that the micro-/nano-structure evolution of bioceramics during the sintering process has a critical effect on their mechanical behavior [40]. For lower cooling rates, there is more time to consolidate the nanostructure that results in lamellae thickening. Furthermore, the microcracks on scaffold surfaces, which are formed by thermal shrinkage during the cooling process, can be reduced by lowering the cooling rate [41]. Accordingly, it is possible to control the micro-/nano-structure and compressive strength of the scaffolds synthesized by this method so that they resemble natural cancellous bone and are adjustable to the requirements of each bone defect.

The in vitro cell test showed that ah-MSCs adhered, spread, and proliferated over scaffold surfaces. High cell colonization was achieved by forming an extensive monolayer on the entire structure, even inside pores. The flattened cell morphology and the large number of exocytosis vesicles were indicators of a good biological response and sound metabolic activity. The Alamar Blue assay (Figure 8) proved that the scaffold’s material stimulated ah-MSCs’ metabolic function. From 28 days in GM and OM the proliferation rate of the ah-MSCs seeded on scaffold surfaces overtook that of the control. This effect was strong under osteogenic conditions as OM was supplemented with β-glycerolphosphate, which is a source of inorganic phosphate that is known to be an important signaling molecule for cell proliferation [42]. Hence, it can be affirmed that the material did not induce cytotoxicity and stimulated cell proliferation.

ALP activity in the ah-MSCs incubated with the scaffold for 28 days was significantly greater than in the control. As ALP is an early marker of osteogenic differentiation, the scaffold’s material could have stimulated the ah-MSCs to differentiate into the osteoblast phenotype. The formation of collagen-like fibrils and some mineral deposits suggest the formation of an extracellular matrix and mineralized nodules. In bone, collagen is present as fine fibers with no preferred orientation, and usually as short segments [43,44] like the collagen-like fibrils in Figure 5F,G. It is at the collagen fibers site where the first HA-crystals grow during the bone biomineralization process [38,45]. As osteoblasts are cells committed to produce collagen fibers on bone growth surfaces, ah-MSCs may have differentiated to the osteoblast phenotype on scaffold surfaces to produce collagen-like fibers. However, more detailed studies of cell differentiation in the presence of scaffolds are needed to corroborate these observations.

When scaffolds were immersed in GM solution, their surface remarkably changed and presented distinct morphologies depending on the immersion time. Different processes took place in the scaffold-GM interphase. At the beginning, individual cells adhered directly to scaffold surfaces. Those areas not covered by cells reacted with the surrounding GM, which led to one of the phases in the alternating lamellae nanostructure to dissolve. According to SEM-EDS, lack of silicon on the scaffold surfaces meant that the dissolved phase was SC. SC phase degradation controlled the creation of a nanoporous reaction zone on scaffold surfaces. The reaction continued internally through the narrow channels within the α-TCP lamellae as SC dissolved (Figure 6). Next, spherical-shaped hydroxyapatite-like precipitation took place on scaffold surfaces. Hydroxyapatite-like precipitation was less copious at the beginning of the reaction, but turned into a heavy dense layer toward the end and covered the entire scaffold surfaces to form a layer-by-layer structure that was closely related to cell membranes (Figure 5I). This layer-by-layer structure could improve the stable chemical bonding between scaffolds and receptor bone tissue.

Si ions, which dissolved from the SC lamellae, were trapped by the new precipitate layer to produce silicon co-substituted hydroxyapatite on the periphery of scaffolds. The EDS data suggested a small quantity of Si substitution in the hydroxyapatite phase according to 59.94-38.56-1.50/Ca-P-Si atm% (Figure 6). The Si substitutions in precipitated hydroxyapatite can stimulate bone cells at the genetic level to promote cell proliferation and differentiation [46,47]. A similar reaction mechanism in simulated body fluid (SBF) has been described for this material in a previous work [48]. The eutectoid scaffold material reacts by dissolving the SC phase and them developing a hydroxyapatite microporous structure by the pseudomorphic transformation of α-TCP lamellae. Next silicon co-substituted hydroxyapatite formed a layer on scaffold surfaces.

The HRTEM and SAD patterns confirmed the hydroxyapatite phase on scaffold surfaces. The apatite crystallization process commenced with distances shorter than 0.300 nm and ended roughly at 0.340 nm, from which crystals grew in line with a preferred (002) orientation (Figure 7E, inner). This preferential orientation is related to hydroxyapatite rearrangement, whose orientation changed and favored preferential growth in new crystals. With time, single crystals started to join and form a dense layer. This crystal coalescence effect has been reported in various calcified tissue formations, referred to as “crystal fusion”, in which a single crystal tends to form from two apatite crystals joining and adhering [33,49].

The inorganic bone component is a calcium-deficient nonstoichiometric hydroxyapatite. It is well-known that the range of Ca/P values depends on many factors like the specimen donor’s gender, age and nutrition, as well as the extraction site. However, the Ca/P ratio for cancellous bone is generally considered to lie between 1.37 < Ca/P < 1.87 [45]. The biomineralization process uses mainly Ca and Si combined with carbonates and phosphates to form biological apatite. The above-described process, which takes place through the nucleation and growth of hydroxyapatite crystals on scaffold surfaces in GM, resembles the natural mechanism of bone mineralization. A similar process to bone mineralization could have taken place in vitro on scaffold structures in the presence of ah-MSCs.

In vitro SBF assays are widely used for in vivo bioactivity prediction purposes. However, some authors [50] have reported that SBF is supersaturated in relation to the hydroxyapatite crystal and forces its precipitation. Consequently, the validity of the SBF assay is questioned because a biomaterial never comes into in situ contact with inorganic fluid. Bioactive behavior not only depends on the material’s composition, but also depends on the solution used for in vitro tests. Some authors have tested the in vitro bioactivity of glass and glass ceramics in DMEM medium (containing similar ionic concentrations as blood plasma, as well as growth factors and proteins present in blood) [51,52]. Although the use of DMEM resulted in the delayed start of precipitation, the precipitated apatite layer was similar to that formed in SBF. This work reports precipitate hydroxyapatite formation in a similar organic environment similar to that of the bone mineral phase. The precipitate did not obstruct cell adhesion and proliferation.

Scaffold functionalization with different molecules and growth factors improves the interaction between the implant and surrounding tissue. In this study, ALP was chosen as the protein model for functionalization because its activity plays an active role in the mineralization process by providing local enrichment of Ca^2+^ and PO_4_^3−^ ions [20,21]. Furthermore, previous studies have supported the correct functionalization of bioactive glasses and glass ceramic with an ALP model [53,54].

The ALP was successfully grafted onto scaffolds, whose enzymatic activity remained until 21 days after functionalization. It should be noted that the ALP amount decreased for 14 and 21 days compared to seven days (Figure 10). There are different types of interaction between proteins and material surfaces: simple adsorption, covalent bonding and electrostatic interaction. Regardless of anchorage type, in this case it can be affected by the washing processes between one test and the next. This may indicate that the ALP could be released to the washing solution. Therefore, more detailed studies are needed to understand the functionalization procedure’s physico-chemical behavior. In any case, the enzyme was grafted onto scaffold structures and its active state remained with no prior surface functionalization treatment, which is already a good result. The presence of the ALP enzyme would support both the biomineralization process and cell activity.

## 5. Conclusions

The micro/nano-structure obtained in the synthesized scaffolds with the 46.6% α-TCP—53.4% SC composition mimics the micro-/nano-structure of natural cancellous bone. The nanostructure consisted of irregular eutectoid lamellae of alternating SC and α-TCP, which were affected by the cooling rate used via the eutectoid temperature. Therefore, it is possible to control the micro-/nano-structure of scaffolds and compressive strength by this sintering process, which would make them similar to natural cancellous bone and they could adjust to the requirements of each bone defect. In supplemented DMEM medium, the SC phase dissolved by modifying scaffolds’ topography, which led to the in-situ formation of the nanoporous structure on surfaces. Later, a dense silicon co-substituted hydroxyapatite precipitated to form a closely related layer-by-layer structure to cell membranes to cover the entire scaffold surfaces. Scaffolds supported the adhesion and proliferation of ah-MSCs, and high colonization was achieved over entire surfaces 28 days after cell seeding. No evidence for cytotoxicity was found, although good metabolic activity was evidenced. ALP anchoring was effectively achieved, and its activity remained after 21 days since grafting. A fraction of the grafted ALP remained attached to scaffolds even after the washing process. The overall results indicate that scaffolds mimicked the structure and chemical composition of natural bone when considering cell biology and biomolecule functionalization. Therefore, the developed scaffold is an interesting material as a bone constructor for tissue engineering.

## Figures and Tables

**Table 1:** Scaffolds’ structural parameters and compressive strength for different cooling rates.

Cooling Rate	50 °C/h	16.5 °C/h	5.5 °C/h
Volumetric Shrinkage (%) *	30 ± 0.5	30 ± 0.5	30 ± 0.5
Compressive strength (MPa) *	0.62 ± 0.07	1.67 ± 0.05	3.38 ± 0.06
Lamellar width range (nm)	100–250	300–560	600–940

* Data represented as mean ± standard deviation.

## Data Availability

Data sharing is not applicable for this paper.

## References

[1] Roseti L., Parisi V., Petretta M., Cavallo C., Desando G., Bartolotti I., Grigolo B. (2017). Scaffolds for bone tissue engineering: State of the art and new perspectives. Mater. Sci. Eng. C.

[2] Jones J.R. (2009). New trends in bioactive scaffolds: The importance of nanostructure. J. Eur. Ceram. Soc..

[3] García J.R., García A.J. (2016). Biomaterial-mediated strategies targeting vascularization for bone repair. Drug Deliv. Transl. Res..

[4] Paredes B., Santana A., Arribas M.I., Vicente-Salar N., de Aza P.N., Roche E., Such J., Reig J.A. (2011). Phenotypic differences during the osteogenic differentiation of single cell-derived clones isolated from human lipoaspirates. J. Tissue Eng. Regen. Med..

[5] Agarwal R., García A.J. (2015). Biomaterial strategies for engineering implants for enhanced osseointegration and bone repair. Adv. Drug Deliv. Rev..

[6] Yu H., Liu K., Zhang F., Wei W., Chen C., Huang Q. (2017). Microstructure and in vitro bioactivity of silicon-substituted hydroxyapatite. Silicon.

[7] Serena S., Caballero A., de Aza P.N., Sainz M.A. (2015). New evaluation of the in vitro response of silicocarnotite monophasic material. Ceram. Int..

[8] Eliaz N., Metoki N. (2017). Calcium Phosphate Bioceramics: A Review of Their History, Structure, Properties, Coating Technologies and Biomedical Applications. Materials.

[9] Kokubo T., Ito S., Huang Z.T., Hayashi T., Sakka S., Kitsugi T., Yamamuro T. (1990). Ca, P-rich layer formed on high-strength bioactive glass-ceramic A-W. J. Biomed. Mater. Res..

[10] Lotsari A., Rajasekharan A.K., Halvarsson M., Andersson M. (2018). Transformation of amorphous calcium phosphate to bone-like apatite. Nat. Commun..

[11] Mate-Sanchez de Val J.E., Calvo-Guirado J.L., Delgado-Ruiz R.A., Ramirez-Fernandez M.P., Negri B., Abboud M., Martinez I.M., de Aza P.N. (2012). Physical Properties, Mechanical Behavior, and Electron Microscopy Study of a New α-TCP Block Graft with Silicon in an Animal Model. J. Biomed. Mater. Res. A.

[12] Wu C., Chang J. (2013). A Review of Bioactive Silicate Ceramics. Biomed. Mater..

[13] Martínez I.M., Velasquez P.A., de Aza P.N. (2012). The sub-system α-TCPss-silicocarnotite within the binary system Ca_3_(PO_4_)_2_–Ca_2_SiO_4_. J. Am. Ceram. Soc..

[14] De Aza P.N., Serena S., Luklinska Z.B. (2018). Manufacture and characterization of a new Si-Ca-P biphasic ceramic. Ceram. Int..

[15] Díaz-Arca A., Mazón P., de Aza P.N. (2019). Eutectoid scaffold as potential tissue engineer guide. J. Am. Ceram. Soc..

[16] McDowell E.M., Trump B.F. (1976). Histologic fixatives suitable for diagnostic light and electron microscopy. Arch. Pathol. Lab. Med..

[17] Meseguer-Olmo L., Aznar-Cervantes S., Mazón P., De Aza P.N. (2012). “In vitro” behavior of adult mesenchymal stem cells of human bone marrow origin seeded on a novel bioactive ceramic in the Ca_2_SiO_4_–Ca_3_(PO_4_)_2_ system. J. Mater. Sci. Mater. Med..

[18] Dominici M., Le Blanc K., Mueller I., Slaper-Cortenbach I., Marini F., Krause D., Deans R., Keating A., Prockop D., Horwitz E. (2006). Minimal criteria for defining multipotent mesenchymal stromal cells. The international society for cellular therapy position statement. Cytotherapy.

[19] De Aza P., García-Bernal D., Cragnolini F., Velasquez P., Meseguer-Olmo L. (2013). The effects of Ca_2_SiO_4_–Ca_3_(PO_4_)_2_ ceramics on adult human mesenchymal stem cell viability, adhesion, proliferation, differentiation and function. Mater. Sci. Eng. C..

[20] Golub E.E., Boesze-Battaglia K. (2007). The role of alkaline phosphatase in mineralization. Curr. Opin. Orthop..

[21] De Jonge L.T., Leuwemburg S.C.G., van den Beucken J.J.J.P., Wolke J.G.C., Jansen A. (2009). Electrosprayed enzyme coatings as bioinspired alternative to bioceramic coatings for orthopedic and oral implants. Adv. Funct. Mater..

[22] Hench L.L. (1991). Bioceramics: From Concept to Clinic. J. Am. Cerm. Soc..

[23] Boyde A., Hobdell M.H. (1968). Scanning Electron Microscopy of Lamellar Bone. Z. Zellforsch..

[24] Boyde A., Hobdell M.H. (1969). Scanning Electron Microscopy of primary membrane bone. Z. Zellforsch..

[25] Jayasinghe J.A.P., Jones S.J., Boyde A. (1993). Scanning electron microscopy of human lumbar vertebral trabecular bone surfaces. Virchows Archiv. A Pathol. Anat..

[26] Martínez I.M., Velasquez P.A., de Aza P.N. (2010). Synthesis and stability of α-tricalcium phosphate doped with dicalcium silicate in the system Ca_3_(PO_4_)_2_–Ca_2_SiO_4_. Mater. Charact..

[27] Ibáñez J., Artús L., Cuscó R., López A., Menéndez E., Andrade M.C. (2001). Hydration and carbonation of monoclinic C_2_S and C_3_S studied by Raman spectroscopy. J. Raman Spectrosc..

[28] Antonakos A., Liarokapis E., Leventouri T. (2007). Micro-Raman and FTIR studies of synthetic and natural apatites. Biomaterials.

[29] Jillavenkatesa A., Condrate R.A. (1998). The infrared and Raman spectra of β-and α tricalcium phosphate (Ca_3_(PO_4_)_2_). Spectrosc. Lett..

[30] De Bruijn J.D., van Blitterswijk C.A., Davies J.E. (1995). Initial bone matrix formation at the hydroxyapatite interface in vivo. J. Biomed. Mater. Res..

[31] Goonoo N., Fahmi A., Jonas U., Gimié F., Arsa I.A., Bénard S., Schönherr H., Bhaw-Luximon A. (2019). Improved Multicellular Response, Biomimetic Mineralization, Angiogenesis, and Reduced Foreign Body Response of Modified Polydioxanone Scaffolds for Skeletal Tissue Regeneration. ACS Appl. Mater. Interfaces.

[32] Liao S., Watari F., Xu G., Ngiam M., Ramakrishna S., Chan C.K. (2007). Morphological Effects of Variant Carbonates in Biomimetic Hydroxyapatite. Mater. Lett..

[33] Dey A., Bomans P.H.H.A., Müller F.A., Will J., Frederik P.M., de With G., Sommerdijk N.A.J.M. (2010). The Role of Prenucleation Clusters in Surface-Induced Calcium Phosphate Crystallization. Nat. Mater..

[34] Porter A.E., Botelho C.M., Lopes M.A., Santos J.D., Best S.M., Bonfield W. (2004). Ultrastructural comparison of dissolution and apatite precipitation on hydroxyapatite and silicon-substituted hydroxyapatite in vitro and in vivo. J. Biomed. Mater. Res. A.

[35] Freyman T.M., Yannas I.V., Gibson L.J. (2001). Cellular materials as porous scaffolds for tissue engineering. Prog. Mater. Sci..

[36] Lu J.X., Flautre B., Anselme K., Hardoiun P., Gallur A., Descamps M., Thierry B. (1999). Role of interconnections in porous bioceramics on bone recolonisation in vitro and in vivo. J. Mater. Sci. Mater. Med..

[37] Hutmacher D.W., Schantz J.T., Lam C.X., Tan K.C., Lim T.C. (2007). State of the art and future directions of scaffold-based bone engineering from a biomaterials perspective. J. Tissue Eng. Regen. Med..

[38] Buckwalter J.A., Glimcher M.J., Cooper R.R., Recker R. (1995). Bone Biology. Part I: Structure, Blood Supply, Cells, Matrix, and Mineralization. J. Bone Jt. Surg. Am..

[39] Stefanescu D.M. (2015). Science and Engineering of Casting Solidification.

[40] Bang L.T., Ishikawa K., Othman R. (2011). Effect of silicon and heat-treatment temperature on the morphology and mechanical properties of silicon—Substituted hydroxyapatite. Ceram. Int..

[41] Kim Y.U., Lee B.H., Kim M.C., Kim K.N., Kim K.M., Choi S.H., Kim C.K., LeGeros R.Z., Lee Y.K. (2006). Effect of Cooling Rate and Particle Size on Compressive Strength of Macroporous Hydroxyapatite. Key Eng. Mater..

[42] Kanatani M., Sugimoto T., Kano J., Chihara K. (2002). IGF-I mediates the stimulatory effect of high phosphate concentration on osteoblastic cell proliferation. J. Cell. Phys..

[43] Jones S.J. (1974). Secretory territories and rate of matrix production of osteoblasts. Calcif. Tissue Res..

[44] Pazzaglia U.E., Congiu T., Marchese M., Dell’Orbo C. (2010). The shape modulation of osteoblast-osteocyte transformation and its correlation with the fibrillar organization in secondary osteons. Cell Tissue Res..

[45] Wu S., Liu X., Yeung K.W.K., Liu C., Yang X. (2014). Biomimetic porous scaffolds for bone tissue engineering. Mat. Sci. Eng. R..

[46] Pietak A.M., Reid J.W., Stott M.J., Sayer M. (2007). Silicon substitution in the calcium phosphate bioceramics. Biomaterials.

[47] Fielding G., Feuerstein J., Bandyopadhyay A., Bose S. (2012). SiO_2_ and SrO Doped β-TCP: Influence of Dopants on Mechanical and Biological Properties. Biomaterials Science: Processing, Properties and Applications II.

[48] Díaz-Arca A., Velasquez P., Mazon P., De Aza P.N. (2020). Mechanism of in vitro reaction of a new scaffold ceramic similar to porous bone. J. Eur. Ceram. Soc..

[49] Rey C., Combes C., Drouet C., Sfihi H., Barroug A. (2007). Physico-Chemical Properties of Nanocrystalline Apatites: Implications for Biominerals and Biomaterials. Mater. Sci. Eng. C.

[50] Bohner M., Lemaitre J. (2009). Can bioactivity be tested in vitro with SBF solution?. Biomaterials.

[51] Lutisanova G., Palou M.T., Kozankova J. (2011). Comparison of bioactivity in vitro of glass and glass ceramic materials during soaking in SBF and DMEM medium. Ceram. Silik..

[52] Theodorou G., Goudouri O.M., Kontonasaki E., Chatzistavrou X., Papadopoulou L., Kantiranis N., Paraskevopoulos K.M. (2011). Comparative Bioactivity Study of 45S5 and 58S Bioglasses in Organic and Inorganic Environment. Bioceram. Dev. Appl..

[53] Verné E., Ferraris S., Vitale-Brovarone C., Spriano S., Bianchi C.L., Naldoni A., Morra M., Cassinelli C. (2010). Alkaline phosphatase grafting on bioactive glasses and glass ceramics. Acta Biomater..

[54] Verné E., Ferraris S., Vitale-Brovarone C., Cochis A., Rimondini L. (2014). Bioactive glass fuctionalized with alkaline phosphatase stimulates bone extracellular matrix deposition and calcification in vitro. Appl. Surf. Sci..

